# The study on the core personality trait words of Chinese medical university students based on social network analysis

**DOI:** 10.1097/MD.0000000000008078

**Published:** 2017-09-15

**Authors:** Ying Wu, Yunzhen Xue, Zhanling Xue

**Affiliations:** School of Humanities and Social Sciences, Shanxi Medical University, Taiyuan, China.

**Keywords:** Chinese medical university students, personality trait words, SNA

## Abstract

The medical university students in China whose school work is relatively heavy and educational system is long are a special professional group. Many students have psychological problems more or less. So, to understand their personality characteristics will provide a scientific basis for the intervention of psychological health.

We selected top 30 personality trait words according to the order of frequency. Additionally, some methods such as social network analysis (SNA) and visualization technology of mapping knowledge domain were used in this study.

Among these core personality trait words *Family conscious* had the 3 highest centralities and possessed the largest core status and influence. From the analysis of core-peripheral structure, we can see polarized core-perpheral structure was quite obvious. From the analysis of K-plex, there were in total 588 “K-2”K-plexs. From the analysis of Principal Components, we selected the 11 principal components.

This study of personality not only can prevent disease, but also provide a scientific basis for students’ psychological healthy education. In addition, we have adopted SNA to pay more attention to the relationship between personality trait words and the connection among personality dimensions. This study may provide the new ideas and methods for the research of personality structure.

## 1 Introduction

The medical university students in China whose school work is relatively heavy and educational system is long are a special professional group. Many students have psychological problems more or less.^[1,2]^ So, to understand their personality characteristics will provide a scientific basis for the intervention of psychological health.

The study on personality is important part in psychology field and the personality words that are basic research tools have been widely used in psychology field. The study from the perspective of the vocabulary dimensions already had a long history in the west dated back to 1000 words list made by Galton. American psychologists Allport and Odbert^[3]^ selected 17,953 terms to distinguish the different human behaviors from Webster's dictionary in 1925 which had provided the necessary foundation for studying personality dimensions from the personality factors vocabulary. Cattel and Eber^[4]^ provided 16PF and Tupes and Christal^[5]^ provided big 5 factors model of the personality and verified by many psychologists.

SNA is a method of sociology which considered society is not made up of individual but of nets and it can analyze the structure and attributive character by the relationship among nodes.^[6,7]^ This study will use SNA to research the core personality trait words of Chinese medical university students.

## 2 Methods

In this study, there were in total 118 personality trait words that came from 5 mature psychological scales: NEO-PI-R, 16PF, CPI, CBF-PI, and QZPS.

We tested 261 medical university new students and everyone should choose 10 words reflecting their personality from the 118 personality trait words. We have taken back 256 scales and rejected 5 scales and the effective rate was 98.1%.

Then, we selected 30 words of high frequency after dealing with the date which was imported into the software excel to form co-occurrence matrix (Table 1). Ucinet, Netdraw, and SPSS were used in SNA.

**Table 1:**
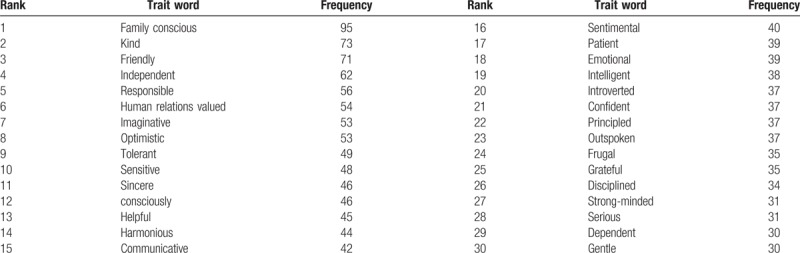
The top 30 personality trait words according to the order of frequency.

Ethical approval was not necessary for this study, because all the data were obtained from Chinese university freshman's psychological census data.

## 3 Results

### 3.1 Analysis of centrality

**Figure 1:**
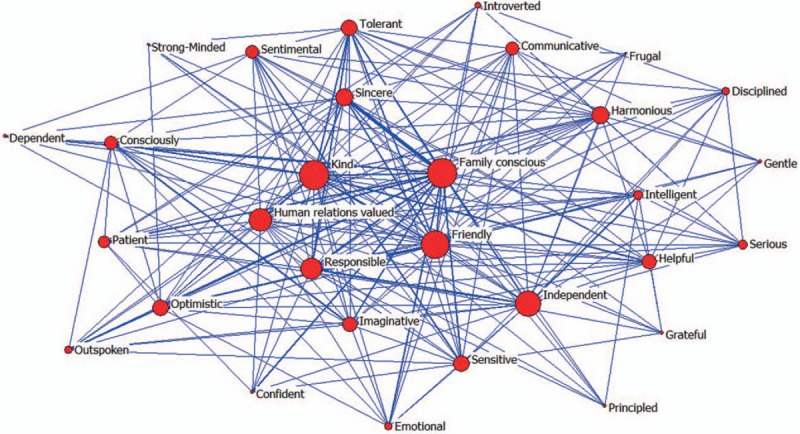
The structure map of 30 core personality trait words.

Figure 1 was the structure map of 30 core personality trait words. The value was bigger between 2 nodes, the occurrence relationship was stronger and the distance is closer. Otherwise, the value was smaller between 2 nodes, the occurrence relationship was weaker and the distance was farther. The size of nodes represented the level of degree centrality. The thickness of the line represented co-occurrence times of each pair of words. The thicker the line, meant the more co-occurrence times.

We ever applied centrality analysis which reflects status and rights of activities in their social network on scientific collaboration in Chinese psychiatry research.^[8]^ There are 3 common centrality measures: degree centrality, betweenness centrality, and closeness centrality.^[9]^ In this network, degree centrality was equal to the number of nodes that connect with a central node. That was, if a word had the highest degree centrality, it was considered a central node in the collaboration network. Betweenness centrality is the number of the shortest paths that pass through a given node.^[10]^ In our study, the highest betweenness centrality would indicate that a word possesses and controls a great deal of research resource. Finally, closeness centrality of a node is equal to reciprocal of the total distance from this node to all other nodes. It meant the closer a node was to all other nodes, the higher is its closeness centrality. The lowest closeness centrality indicated a word was at the core position of the entire network.

In this network, we selected the top 10 words with the highest centrality in this study (Table 2). These 10 words that were strong in terms of power, prestige, and influence were the core position in this network. Among these words, *Family conscious* which had the 3 highest centralities possessed the largest core status and influence.

**Table 2:**
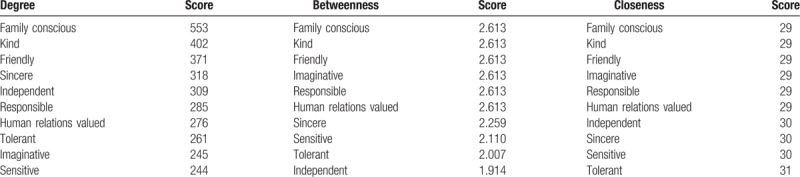
The top 10 words centrality measures in words network.

### 3.2 Analysis of core-peripheral structure

**Figure 2:**
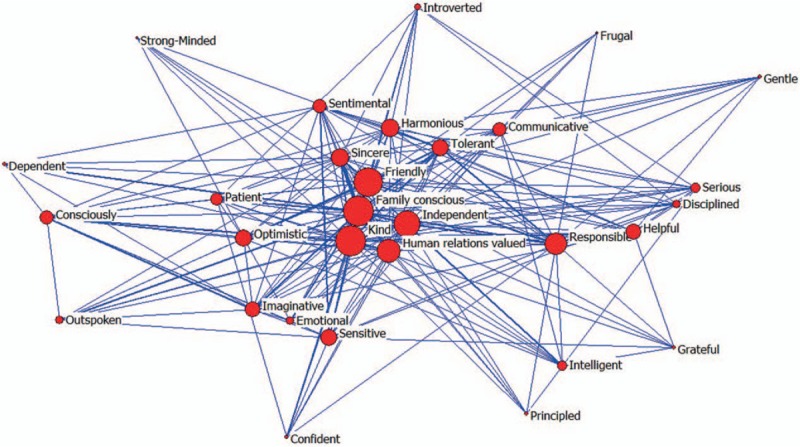
The core-periphery structure map of 30 core personality trait words.

The analysis of core-peripheral structure which distinguishes a series of actors with higher densities (core) and lower densities (peripheral) is the quantitative research on all the networks. The core actors are in a dominant within the exchanging relationship of peripheral actors.^[11]^ We applied this method into the social network analysis of the core personality trait words and found that correlative value was 0.712 (Ucinet 6.0). From Fig. 2, we can see polarized core-peripheral structure was quite obvious. The 30 personality trait words were divided into core words and peripheral words. The core words were *Family conscious*, *Kind*, *Friendly*, *Independent*, *Responsible*, *Human relations valued*, *Imaginative*, *Optimistic*, *Tolerant*, *Sensitive*, *Consciously,* and *Helpful*. The peripheral words were *Sincere*, *Helpful*, *Harmonious*, *Communicative*, *Sentimental*, *Patient*, *Emotional*, *Intelligent*, *Introverted*, *Confident*, *Principled*, *Outspoken*, *Frugal*, *Grateful*, *Disciplined*, *Strong-minded serious*, *Dependent,* and *Gentle*.

### 3.3 Analysis of K-plex

**Figure 3:**
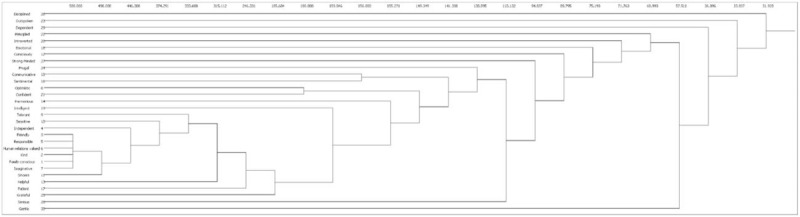
The K-plex structure of 30 core personality trait words.

A K-plex is a maximal subnetwork in which each node is at least connected with other nodes except these K nodes directly within the subnetwork.^[12]^ In the first, determine the condensation degree of subgroup. To exclude the phenomenon of the fewer number of collaboration, “k” was determined to be ”2.” It indicated the members connected with others n − 2 times and these subgroups were higher cohesive in which the members were keeping a relative close relationship. If there were 5 members in the first K-plex, there were 3 numbers connecting with others. In this study, if the value of k was 2, there were in total 588 2-plexs, among which there were factors that are *Family conscious*, *Kind*, *Friendly*, *Responsible*, *Human relations valued,* and *Imaginative* in 2-plexs by using Ucinet (Fig. 3).

### 3.4 Analysis of principal components

**Figure 4:**
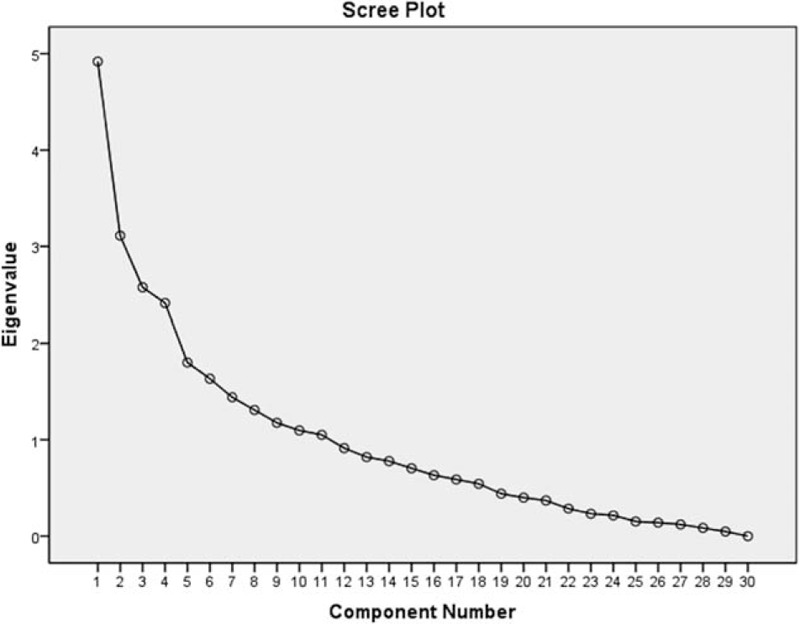
The scree plot of principal components analysis.

Principal components analysis (PCA) is a mutidimensional factor included in the same system for qualitative and quantitative research which is relatively perfect multivariate statistical analysis methods. PCA takes out several comprehensive variables according to the actual need to reflect the original information and simplify the data. In this study, we introduce the co-occurrence matrix of 30 personality traits words into SPSS 19.0, and then run the principal component analysis, principal components sorting according to the personality traits in descending order of frequency. Eigenvalues, Variance, and Cumulative were respectively calculated (Table 3). Based on the Eigenvalues greater than 1, we selected the 11 principal components and the cumulative was 75.08% (Fig. 4). The 11 principal components were *Family conscious, Kind, Friendly, Independent, Responsibility, Human relations valued, Imaginative, Optimistic, Tolerant, Sensitive, and Sincere.*

**Table 3:**
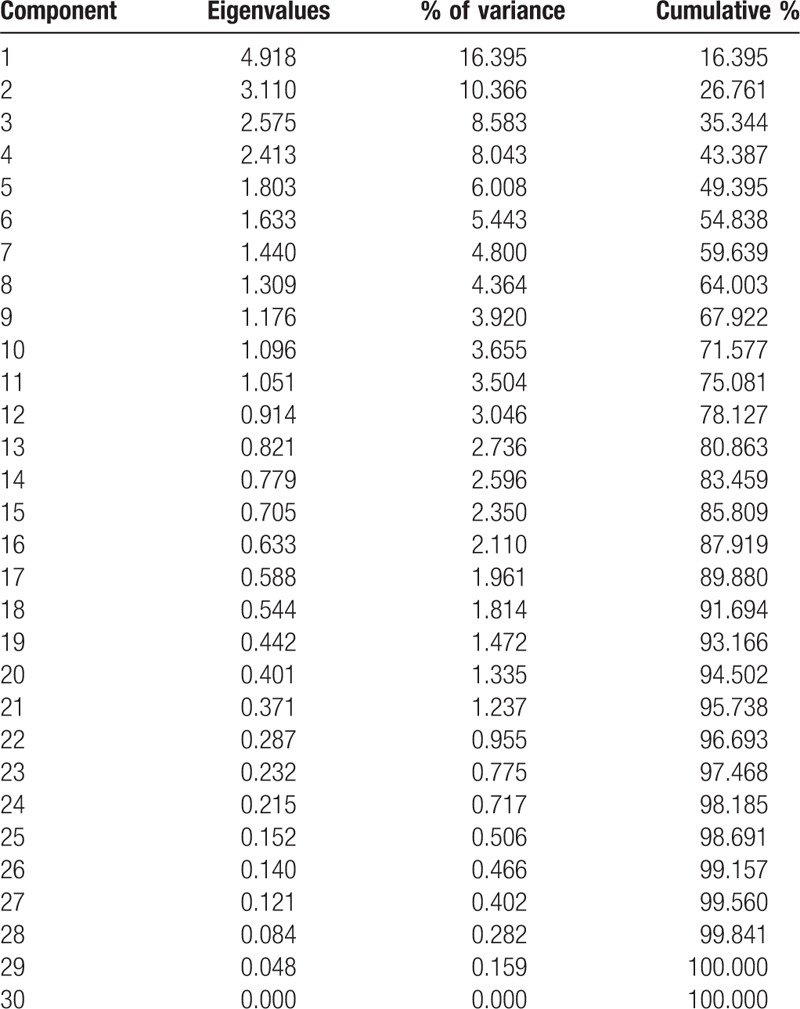
Total variance explained of principal components analysis.

## 4 Discussion

This study applied SNA into the personality trait words from the analysis of centrality, core-peripheral structure, K-plex, and Principal Components. In these core personality trait words, *Family conscious, Kind, Friendly, Responsibility, Human relations valued, and Imaginative* were in the core position in 4 analyses above. Three words were *Independent, Tolerant, and Sensitive* in the core position of the analysis on centrality and core-peripheral. Only *Tolerant* and *Sensitive* were in the core position of the analysis on centrality. Only *Sincere* was in the core position of the analysis on core-peripheral structure. *Independent, Optimistic, Tolerant, Sensitive, and Sincere* were in the core position of the PCA.

From the analysis of these core personality trait words, we could learn that medical university students paid attention to human communication and embodied cultural characteristics relying on the relationship first. It probably related with Chinese harmony and the way of relationship. Second, moral sense as a quality of Chinese self-improvement has been paid great attention by society and medical university students also paid attention to enhance their self-cultivation. Third, the responsibility as an important work personality trait also brought to the attention of the medical university students, but the existence of negative words suggested the responsibility levels retrained to be further improved. At last, because medical university students have not entered society, they were unavoidably idealized for their own development and sometimes their emotion was unstable. It conformed to the young personality characteristics. This study not only contained the commonness of human personality, but also reflected the unique personality factor of Chinese medical university students.

Personality was all essence of individual behavior and bad personality traits were the risk factors of the psychological disease. So, the study of personality not only can prevent disease, but also provide a scientific basis for students’ psychological healthy education.

In addition, the previous studies on the personality structure mostly focused on the research of the personality dimensions and personality functional assessment questionnaire. In this study, we have adopted SNA to pay more attention to the relationship between personality trait words and the connection between personality dimensions. This study may provide the new ideas and methods for the research of personality structure.

## Acknowledgments

The authors thank the participants for their time and willingness to participate in this study. The authors also acknowledge the support for this study from ‘Analysis on Scientific Collaboration and Trends Prediction of Research Fronts in Psychiatry Field (No. 71503152),’ awarded by National Natural Science Foundation of China. This study was also supported by Program for the Outstanding Innovative Teams of Higher Learning Institutions of Shanxi (OIT).
